# Functional interplay between YY1 and CARM1 promotes oral carcinogenesis

**DOI:** 10.18632/oncotarget.26984

**Published:** 2019-06-04

**Authors:** Amit K. Behera, Manoj Kumar, Muthu K. Shanmugam, Aditya Bhattacharya, Vinay J. Rao, Akshay Bhat, Madavan Vasudevan, Kodaganur S. Gopinath, Azeem Mohiyuddin, Anupam Chatterjee, Gautam Sethi, Tapas K. Kundu

**Affiliations:** ^1^ Transcription and Disease Laboratory, Molecular Biology and Genetics Unit, Jawaharlal Nehru Centre for Advanced Scientific Research, Bangalore 560064, India; ^2^ Department of Pharmacology, Yong Loo Lin School of Medicine, National University of Singapore, Singapore 117600, Singapore; ^3^ Bionivid Technology Private Limited, Kasturi Nagar, Bangalore 560043, India; ^4^ Department of Surgical Oncology, HCG Bangalore Institute of Oncology, Bangalore 560027, India; ^5^ Department of Pathology, Sri Devaraj Urs Academy of Higher Education and Research Center, Kolar, Bangalore 563101, India; ^6^ Department of Ear, Nose and Throat, Sri Devaraj Urs Academy of Higher Education and Research Center, Kolar, Bangalore 563101, India; ^7^ Department of Head and Neck Surgery, Sri Devaraj Urs Academy of Higher Education and Research Center, Kolar, Bangalore 563101, India; ^8^ Department of Biotechnology and Bioinformatics, North-Eastern Hill University, Shillong, Meghalaya 793022, India

**Keywords:** oral cancer, oncogene, YY1, CARM1, arginine methylation

## Abstract

Coactivator associated arginine methyltransferase 1 (CARM1) has been functionally implicated in maintenance of pluripotency, cellular differentiation and tumorigenesis; where it plays regulatory roles by virtue of its ability to coactivate transcription as well as to modulate protein function as an arginine methyltransferase. Previous studies establish an oncogenic function of CARM1 in the context of colorectal and breast cancer, which correlate to its overexpressed condition. However, the mechanism behind its deregulated expression in the context of cancer has not been addressed before. In the present study we uncover an oncogenic function of CARM1 in the context of oral cancer, where it was found to be overexpressed. We also identify YY1 to be a positive regulator of CARM1 gene promoter, where silencing of YY1 in oral cancer cell line could lead to reduction in expression of CARM1. In this context, YY1 showed concomitant overexpression in oral cancer patient samples compared to adjacent normal tissue. Cell line based experiments as well as xenograft study revealed pro-neoplastic functions of YY1 in oral cancer. Transcriptomics analysis as well as qRT-PCR validation clearly indicated pro-proliferative, pro-angiogenic and pro-metastatic role of YY1 in oral cancer. We also show that YY1 is a substrate of CARM1 mediated arginine methylation, where the latter could coactivate YY1 mediated reporter gene activation *in vivo*. Taken together, CARM1 and YY1 were found to regulate each other in a positive feedback loop to facilitate oral cancer progression.

## INTRODUCTION

YY1 regulates expression of a wide range of genes involved in multitude of cellular processes such as proliferation, differentiation and apoptosis [1]. YY1 is known to integrate several bio-molecular pathways in cell; therefore its role in patho-physiological manifestation of different human diseases, especially in cancer, has been extensively studied [2]. Its expression has been correlated with malignant phenotype of tumor with prognostic significance in several cancer types [3, 4]. However, in certain forms of cancer, YY1 expression shows unfavorable association with tumorigenesis [5]. Existence of such conflicting scientific evidences indicates a very complex network of genes regulated by YY1, where fine balance among them in different conditions can dictate the outcome [6, 7].

Several studies suggest both oncogenic and tumor suppressor function of YY1 in breast cancer progression. According to one report, YY1 is overexpressed in breast cancer tissue and its knock-down in breast cancer cell lines leads to reduction of clonogenicity, migration and invasion of cells. At molecular level, YY1 was found to exert oncogenic function via negative regulation of p27 expression [8]. Another study documents observations which are completely opposite in nature. According to this study YY1 is able to transactivate expression of tumor suppressor BRCA1 via promoter regulation. Ectopic overexpression of YY1 in breast cancer cell line MDA-MB-231 led to cell cycle arrest and reduced cellular growth *in vitro* and *in vivo*. Clinical investigation also showed negligible expression status of YY1 in breast tumor tissue compared to normal counterpart; which is in direct contradiction to the observation made in the previous study [9]. Tumor suppressor role of YY1 also has been shown in the context of pancreatic ductal adenocarcinoma (PDAC), where YY1 overexpression was found to suppress proliferation, migration, invasion and metastasis of PDAC cells [10–12]. Substantial overexpression of YY1 has been observed in gastro-intestinal cancers [13]. YY1 was found to be overexpressed in esophageal squamous cell carcinoma (ESCC) and its expression correlated with progression and invasiveness of esophageal cancer [14]. YY1 has been found to be overexpressed in melanoma compared to benign nevi and normal tissue control with positive correlation with metastasis and tumor stage [15]. Silencing of YY1 was shown to inhibit proliferation, migration and invasion of melanoma cells. The tumorigenic potential of YY1 in colon cancer has been demonstrated with both overexpression and knock-down experiments in colorectal cancer cell lines such as HCT116, LOVO and DLD [16]. Silencing of YY1 resulted in reduced proliferation and induction of apoptosis, whereas overexpression enhanced proliferation and inhibited apoptosis of colorectal cancer cells. The growth of xenograft tumor tissue in nude mice responded accordingly. At molecular level, YY1 mainly inhibited p53 and activated Wnt signaling pathway to promote colon carcinogenesis [16]. There are multiple reports which unequivocally show upregulation of YY1 in several prostate cancer cell lines as well as prostatic neoplasia of patients [17, 18]. YY1 positively regulates expression of PSCA (Prostate stem cell antigen) via which it is believed to contribute to disease progression and metastasis in prostate cancer [19]. Additionally, YY1 represses expression of death receptor 5 (DR5) and Fas, thereby conferring resistance to TRAIL and FasL induced apoptosis in prostate cancer cells [20–22]. Similar investigations show upregulated expression of YY1 in several other cancer types such as lung cancer, ovarian cancer, cervical cancer, liver cancer, bladder cancer, bone cancer, skin cancer and in a few types of non-solid tumors such as acute myeloid leukemia (AML), follicular lymphoma (FL) and diffuse large B-cell lymphoma (DLBCL) [3, 4]. However, there are no reports on the expression status and functional properties of YY1 in the context of oral cancer. In this study we demonstrate overexpression status of YY1 with oncogenic functions in oral cancer. We also identify CARM1 (Coactivator associated arginine methyltransferase 1) as a coactivator of YY1 mediated transcription *in cellulo*.

CARM1 has been found to be overexpressed in several cancer types such as breast cancer, colorectal cancer and prostate cancer, where it exhibits oncogenic properties [23–29]. However, the role of CARM1 in progression of oral carcinogenesis has not been explored before. Oral cancer is one of the major causes of mortality in several countries of the world [30, 31]. It has gained huge scientific interest in last decade as a major health issue in Melanesia, South-central Asia and Central and Eastern Europe [30]. Several studies carried out by multiple groups have established the correlation between etiology and clinical manifestation of oral cancer, facilitating development of preventive measures [32, 33]. However, extensive molecular biology research would be necessary to identify different oncogenic candidates so that chemotherapy specific for oral cancer could be designed. Keeping in mind the need of the hour, we put our effort in the present study to elucidate the molecular function of CARM1, a prospective oncogenic candidate and a probable therapeutic target in the context of oral cancer. In this report, we uncover interplay between YY1 and CARM1 that promotes oral carcinogenesis.

## RESULTS

### CARM1 is overexpressed in oral cancer

**Figure 1:**
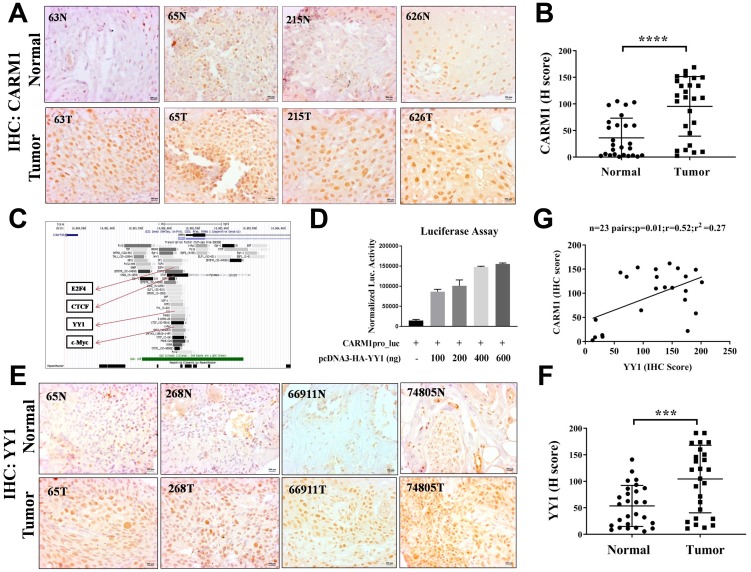
CARM1 and YY1 are overexpressed in oral cancer patient tumor tissue. (**A**) Representative immunohistochemistry images of paired oral cancer patient samples stained with CARM1 antibody. The numbers on images represent patient ID (e.g., 63, 65, 215 and 626). Image scale: 500 μm. (**B**) H-scoring for CARM1 staining in oral cancer patient tumor tissue compared to adjacent normal tissue (*n =* 26; Student’s *t*-test: ^****^p < 0.0001). (**C**) Snapshot of Genome browser from ENCODE to show enrichment of different transcription factors on CARM1 promoter. Highlighted are E2F4, CTCF, YY1 and c-Myc. (**D**) Luciferase assay with transfection of increasing amounts of pcDNA3-HA-YY1 (e.g., 100ng, 200ng, 400ng, 600ng) in HEK293T cells in a dose dependent manner (*n =* 2). (**E**) Representative immunohistochemistry images of paired oral cancer patient samples stained with YY1 antibody. The numbers on images represent patient ID (e.g., 65, 268, 66911 and 74805). Image scale: 500 μm. (**F**) H-scoring for YY1 staining in oral cancer patient tumor tissue compared to adjacent normal tissue (*n =* 27 and ^***^p < 0.001). (**G**) Correlation analysis of H-scores of CARM1 and YY1 immunohistochemistry in oral tumor tissues (*n =* 23, *p =* 0.01, *r =* 0.52, *r*^2^ = 0.27).

We were interested to investigate the expression status of CARM1 in oral cancer patient samples. Immunohistochemistry analysis using CARM1 specific antibody revealed significant upregulation of CARM1 expression in oral cancer tumor tissue compared to normal counterpart (Figure 1A). The H-scoring values of immunostaining for 26 pairs of oral cancer patient samples have been compared in Figure 1B. Immunohistochemistry experiment with H3R17me2a modification also indicated higher prevalence of the mark in tumor samples in comparison with adjacent normal tissue (Supplementary Figure 1A, 1B). As asymmetric dimethylation of H3R17 is specifically mediated by CARM1 enzyme among the PRMT family members, its higher prevalence justifiably could be attributed to CARM1 over-expression. The clinicopathological information has been summarized in Supplementary Table 1. We wanted to further investigate the driving force behind transcriptional upregulation of CARM1 in oral cancer. The genome wide transcription factor enrichment profile from ENCODE repository revealed a few prospective TF candidates for regulation of CARM1 expression in oral cancer such as E2F4, CTCF, YY1 and cMyc etc. (Figure 1C). Bioinformatic prediction for the presence of binding sites of different transcription factors on CARM1 promoter by Consite also confirmed the presence of motifs responsive to above TFs (data not shown). Initial screening with above mentioned transcription factors in regulating CARM1 promoter driven luciferase reporter gene expression revealed a positive regulation of CARM1 promoter by YY1, where transfection of increasing amounts of pcDNA3-HA-YY1 in HEK293T cells enhanced CARM1 promoter activity in a dose dependent manner (Figure 1D).

### YY1 is overexpressed in oral cancer and regulates CARM1 expression

**Figure 2:**
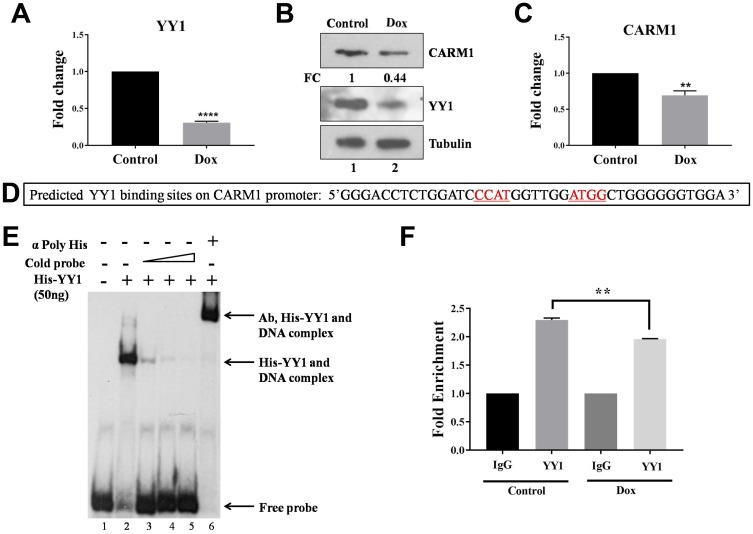
YY1 regulates CARM1 expression. (**A**) qRT-PCR to assess RNA expression of YY1 in AW8507_Tet-ON-shYY1 cells with Doxycycline treatment (*n =* 3, ^***^p < 0.001). (**B**) Immunoblotting to analyze protein expression of YY1 and CARM1 in AW8507_Tet-ON-shYY1 cells with Doxycycline treatment (FC: Fold change). Data is representative of three independent experiments. (**C**) qRT-PCR to assess RNA expression of CARM1 in AW8507_Tet-ON-shYY1 cells with inducible silencing of YY1 (*n =* 3, ^*^p < 0.05). (**D**) The sequence of the probe taken from CARM1 promoter for EMSA. Putative YY1 binding sites have been highlighted. (**E**) EMSA with recombinant full length His-YY1 and radiolabelled oligonucleotide taken from CARM1 promoter. (**F**) ChIP to assess recruitment of YY1 on CARM1 promoter in AW8507_Tet-ON-shYY1 cells (*n =* 3, ^**^p < 0.01, Dox: Doxycycline).

As our results clearly suggest that YY1 positively regulates CARM1 promoter and CARM1 is highly overexpressed in oral cancer patient samples, we wanted to find out whether CARM1 overexpression is caused by upregulation of YY1 in the context of oral cancer. To assess expression status of YY1 in oral cancer, immunohistochemistry (IHC) was performed using YY1 specific antibody. The representative IHC images have been shown in Figure 1E. H-scoring with 27 pairs of oral cancer patient samples revealed significant upregulation of YY1 in oral cancer tumor tissue compared to adjacent normal tissue (Figure 1F). A positive correlation between H-scores for CARM1 and YY1 expression in oral tumor samples was observed (Figure 1G). This finding indicates that CARM1 overexpression could be attributed to have occurred partly due to upregulated expression of YY1. Furthermore, we performed mRNA expression analysis with knock-down of YY1 in a grade III, oral cancer cell line, AW8507. The inducible knock-down of YY1 in AW8507_Tet-ON-shYY1 cells with Doxycycline treatment was found to lead to reduction in expression of CARM1, indicating transcriptional regulation by YY1 in the context of oral cancer (Figure 2A–2C). In order to transcriptionally upregulate CARM1 gene expression, YY1 must bind to the responsive elements on the CARM1 promoter. Consite, an online bioinformatic tool, identified multiple putative binding sites of YY1 on human CARM1 promoter with 80% cut off score (data not shown). One of the high scoring predicted binding sites was selected for validation and oligonucleotide containing YY1 responsive elements was used to perform *in vitro* EMSA (Figure 2D). His-YY1 formed a complex with radiolabelled oligonucleotide that showed decreased intensity when unlabelled probe was allowed to compete for interaction. Supershift of protein-DNA complex was observed in the presence of antibody against the His-tag, further indicating specificity of the complex (Figure 2E). Chromatin immunoprecipitation assay with YY1 antibody suggested recruitment of YY1 on CARM1 promoter, which showed decrease in enrichment upon inducible silencing of YY1 in AW8507_Tet-ON-shYY1 cells (Figure 2F). Taken together, these data prove that YY1 positively regulates CARM1 expression and is at least partly responsible for the overexpression of CARM1 in oral cancer patient tumors.

We also analyzed RNA-seq data available in TCGA (The Cancer Genome Atlas) to determine any possible correlations in RNA expression of CARM1 and YY1 in different cancer types. The expression patterns of the two genes were found to vary across different tumor types (Supplementary Figures 2 and 3). Analysis of correlation status across the different tumor types revealed that the two genes do not follow any particular pattern universally. It was seen that most cancer types, for the particular cohorts showed negative or no correlation, while only ESO and TGCT showed a positive correlation in the expression pattern (Supplementary Figure 4, Supplementary Table 2). Given that only RNA expression data has been compared, and not the protein profiles, it is also possible that post-translational modifications may play a role in protein stability and that the correlation data in such cases may change.

### YY1 and CARM1 exhibit oncogenic function in oral cancer

**Figure 3:**
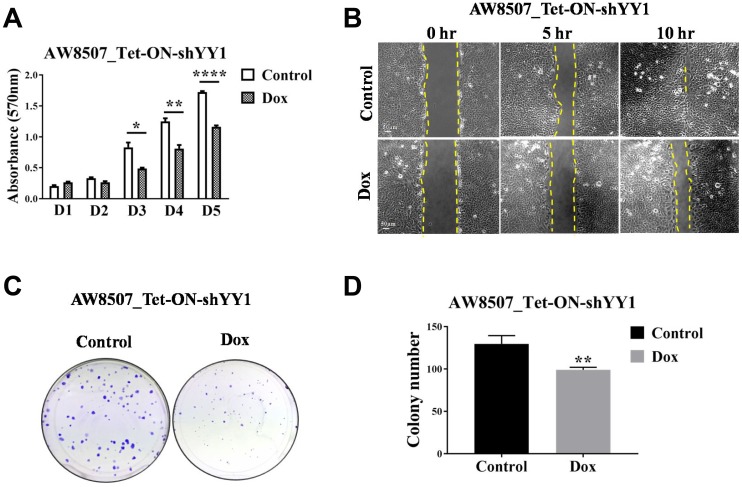
Elucidation of oncogenic role of YY1 in oral cancer cell line. (**A**) MTT assay with AW8507_Tet-ON-shYY1 cells with inducible silencing of YY1 expression (*n =* 3, ^*^p < 0.05, ^**^p < 0.01, ^****^p < 0.0001, D1-D5:Day1-Day5). (**B**) *In vitro* wound healing assay with AW8507_Tet-ON-shYY1 cells with inducible silencing of YY1 expression. Image scale: 50 μm. Data is representative of three independent experiments. (**C**) Clonogenic assay with AW8507_Tet-ON-shYY1 cells with inducible silencing of YY1 expression. (**D**) Quantitation of colony number (*n =* 3, ^**^p < 0.01).

In order to understand the functional significance of YY1 overexpression in oral cancer, different tumorigenic assays were performed in AW8507_Tet-ON-shYY1 stable cell line with inducible silencing of YY1 expression. MTT assay was performed to understand the contribution of YY1 in cellular proliferation. AW8507 cells showed reduced proliferation when YY1 expression was silenced, indicating a pro-proliferative role of YY1 in oral cancer (Figure 3A). Similarly, AW8507 cells with inducible knock-down of YY1 showed slower migration in *in vitro* wound healing assay (Figure 3B). In clonogenic assay AW8507 cells formed fewer and smaller colonies under YY1 silencing conditions, indicating a necessity of YY1 for clonal propagation of oral cancer cells (Figure 3C, 3D). The smaller colonies imply reduction in proliferation ability of cells, further confirming MTT assay results. The findings on wound healing assay and colony formation assay indicate a positive role of YY1 as a pro-metastatic regulator, which might favor EMT (Epithelial to mesenchymal transition) *in vivo*.

**Figure 4:**
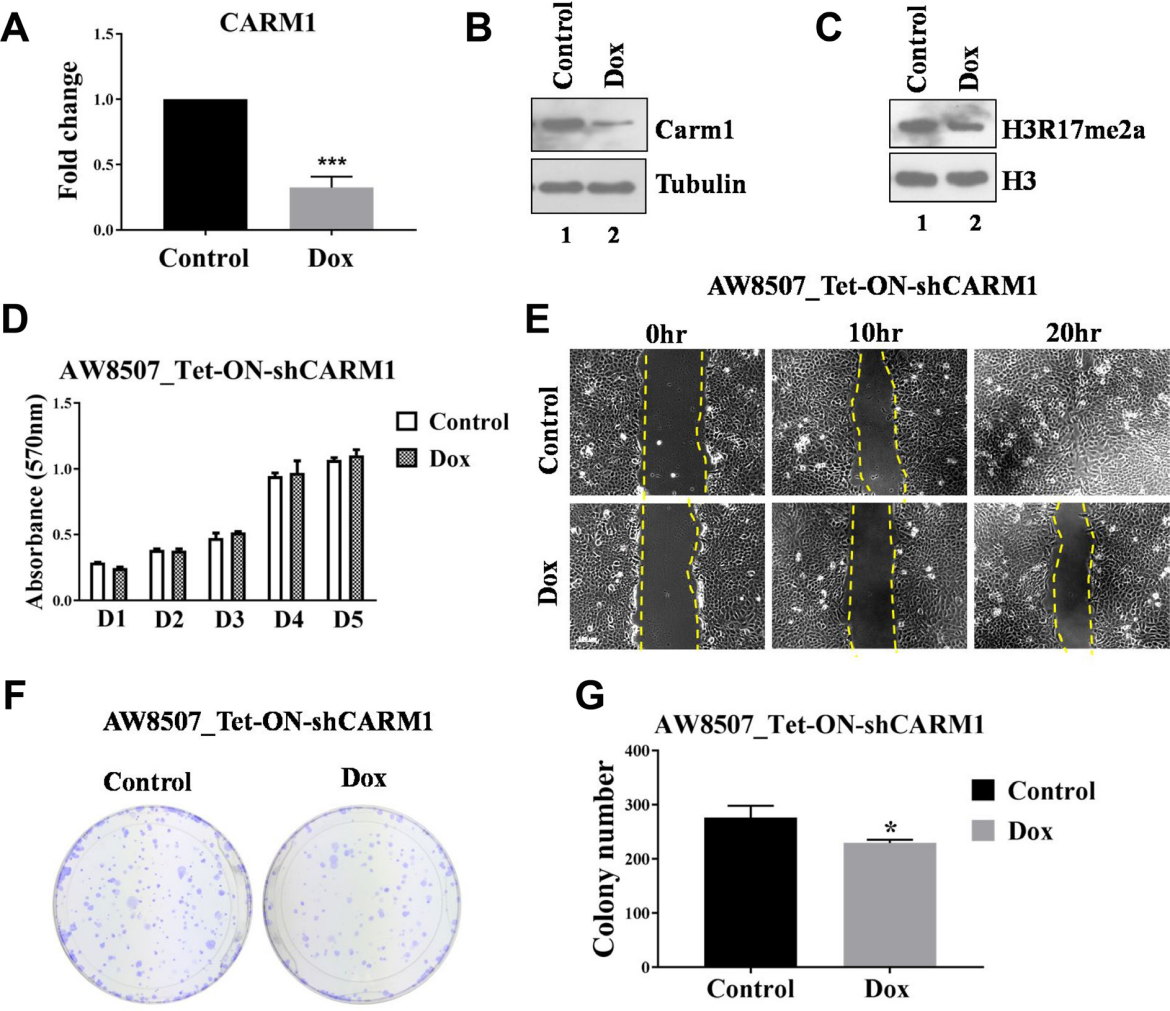
Elucidation of oncogenic role of CARM1 in oral cancer cell line. (**A**) qRT-PCR to assess RNA expression of CARM1 in AW8507_Tet-ON-shCARM1 cells with Doxycycline treatment (*n =* 3, ^***^p < 0.001). (**B**) Immunoblotting to assess protein expression of CARM1 in AW8507_Tet-ON-shCARM1 cells with Doxycycline treatment. Data is representative of three independent experiments. (**C**) Immunoblotting to assess levels of H3R17me2a upon inducible silencing of CARM1 expression in AW8507_Tet-ON-shCARM1 cells. Data is representative of three independent experiments. (**D**) MTT assay with AW8507_Tet-ON-shCARM1 cells with inducible silencing of CARM1 expression (D1-D5:Day1-Day5). (**E**) *In vitro* wound healing assay with AW8507_Tet-ON-shCARM1 cells with inducible knock-down of CARM1 expression. Image scale: 100 μm. Data is representative of three independent experiments. (**F**) Clonogenic assay with AW8507_Tet-ON-shCARM1 cells in presence of Doxyxycline. (**G**) Quantitation of colony number (*n =* 3, ^*^p < 0.05).

Tumorigenic potential of CARM1 was also assessed with inducible silencing of CARM1 in AW8507 cells (Figure 4A–4C). MTT assay was performed to assess proliferation ability of AW8507_Tet-ON-shCARM1 stable cells with inducible silencing of CARM1 expression. However, no change in proliferation was observed in cells with reduced CARM1 expression compared with control cells (Figure 4D), indicating a non-essential role for CARM1 in proliferation. Similarly, *in vitro* wound healing assay and clonogenic assays were performed with inducible knock-down of CARM1 expression. The effect of inducible silencing of CARM1 on migration potential of AW8507_Tet-ON-shCARM1 cells was significant (Figure 4E). In clonogenic assay, the colony establishment capacity was adversely affected in absence of CARM1 as reflected by the difference in colony number (Figure 4F, 4G). However, the colony size was similar in nature, indicating similar proliferation rates irrespective of expression status of CARM1 in AW8507 cells, further confirming the results of MTT assay.

### Determination of gene expression profile modulated by YY1 in oral carcinogenesis

**Figure 5:**
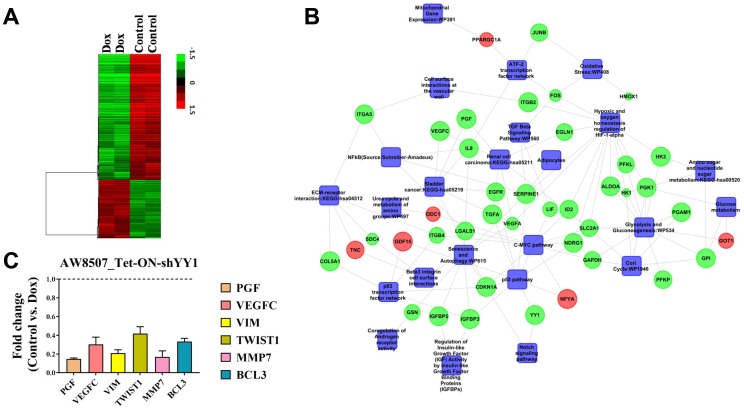
YY1 regulates multiple oncogenic pathways in oral cancer cells. (**A**) Array correlation heat map to show differential gene expression with a fold change value of 1.5 above the median expression value with hierarchical clustering from microarray performed in AW8507_Tet-ON-shYY1 cells. (**B**) A molecular network with integration of multiple pathways which were modulated upon inducible silencing of YY1. Green indicates downregulation, Red indicates upregulation. (**C**) qRT-PCR experiment to assess the expression of various genes related to cancer upon inducible silencing of YY1 in AW8507_Tet-ON-shYY1 cells (*n =* 3). Genes tested were PGF, VEGFC, VIM, TWIST1, MMP7 and BCL3.

From the above experiments, YY1 was revealed to act as an oncogene in the context of oral cancer. We wanted to investigate and determine the gene signature influenced by YY1 to contribute to oral cancer progression. Therefore, microarray based transcriptomic analysis was performed to identify the genes directly or indirectly influenced by YY1 in oral cancer. The differential gene expression data showed up-regulation of 236 transcripts and down-regulation of 510 transcripts with a fold change of ≥1.5 and *p* value of ≤0.05. The hierarchical clustering of differentially expressed genes from biological duplicates of samples has been represented in heat map form in Figure 5A.

Biological pathway enrichment analysis with differentially expressed genes revealed various categories related to proliferation, metabolism, hypoxia and senescence, which have been represented in the form of a molecular network in Figure 5B. Salient features of the network are as follows: (1) Inducible knock-down of YY1 led to suppression of mitogenic pathways such as TGF signaling (both TGFA and TGFB) and c-Myc pathway, which indicates role of YY1 in proliferation of oral cancer cells. (2) Inducible knock-down of YY1 resulted in significant reduction in expression of angiogenesis related factors such as PGF, VEGFC and VEGFR3 etc., which indicates pro-angiogenic role of YY1. (3) Silencing of YY1 also resulted in abrogation of hypoxic signaling pathway and reduction in MMP7 expression, which strongly indicates a putative role of YY1 in regulating metastasis and invasion of oral cancer cells. qRT-PCR validation results showed significant reduction in expression of both PGF and VEGFC upon inducible silencing of YY1. Similarly, expression of genes involved in EMT as well as metastasis such as VIM, TWIST and MMP7 were reduced upon silencing of YY1 in qRT-PCR experiment, indicating a regulatory role of YY1 in metastasis and invasion of oral cancer (Figure 5C). YY1 is known to positively regulate expression of cMyc, VEGF and Vimentin (VIM) genes through promoter activation from previous works of other groups [34–36]. Therefore, the findings of the present study are consistent with the previous reports and further corroborate the role of YY1 in modulating genes relevant in the context of cancer progression.

### Inducible silencing of YY1 impairs tumor growth in mice

**Figure 6:**
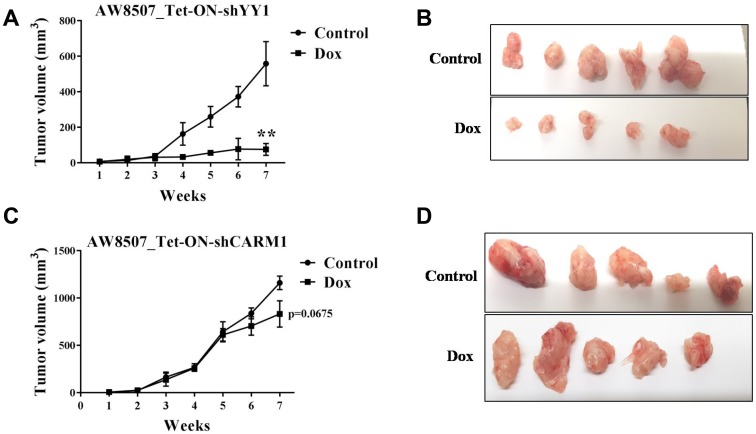
Xenograft study in nude mice with inducible silencing of YY1 and CARM1 in oral cancer cells. (**A**) Xenograft study with AW8507_Tet-ON-shYY1 cells (*n =* 5, ^**^p < 0.01). (**B**) Images of dissected tumors of AW8507_Tet-ON-shYY1 cells after 7 weeks of growth. (**C**) Xenograft study with AW8507_Tet-ON-shCARM1 cells (*n =* 5, *p =* 0.0675). (**D**) Images of dissected tumors of AW8507_Tet-ON-shCARM1 cells after 7 weeks of growth.

In order to understand the role of CARM1 and YY1 in tumor growth *in vivo*, xenograft studies were performed in NSG (NOD-SCID gamma) mice. AW8507_Tet-ON-shYY1 cells showed a very prominent reduction in tumor growth upon inducible knock down of YY1 with Doxycycline treatment (Figure 6A, 6B). However, silencing of CARM1 did not result in as much change in tumor size as was seen with YY1 knock-down (Figure 6C, 6D). In all cases, the body weight of mice remained unaffected by Doxycycline treatment throughout the experiment (Data not shown). The results of xenograft studies further corroborate the *in vitro* cellular data, emphasizing on the important role of YY1 in oral cancer growth.

### CARM1 methylates YY1 and coactivates YY1 mediated transcription

**Figure 7:**
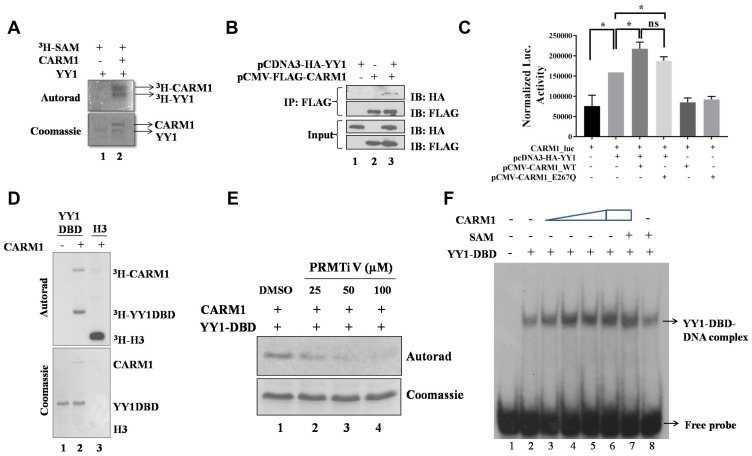
CARM1 methylates YY1. (**A**) *In vitro* methylation assay with recombinant full length His-YY1 and FLAG-CARM1 in presence of tritiated SAM. (**B**) Immuno-pull down assay to assess *in vivo* interaction between YY1 and CARM1 (IP: Immunopulldown and IB: Immunoblotting). (**C**) Reporter luciferase assay to assess coactivation potential of CARM1 in the context of YY1 driven gene expression (*n =* 2, ^*^p < 0.05). (**D**) *In vitro* methylation assay with recombinant His-YY1-DBD and FLAG-CARM1 in presence of tritiated SAM. (**E**) *In vitro* methylation assay with His-YY1-DBD and FLAG-CARM1 in presence of tritiated SAM and PRMT inhibitor V (25, 50 and 100 μM). (**F**) EMSA to determine effect of arginine methylation on DNA binding ability of YY1-DBD (15 minutes methylation assay + 15 minutes DNA binding assay).

Experimental evidences of the present study so far indicated a positive role of YY1 in regulation of CARM1 gene expression. Given the understanding that CARM1 is an arginine methyltransferase and known to methylate multiple transcription factors in cell, it was speculated that YY1 may also undergo arginine methylation. To test whether YY1 is a substrate of CARM1, *in vitro* methylation assays were performed with FLAG-CARM1 and His-YY1 in the presence of tritiated SAM and the samples were processed for autoradiography. CARM1 seemed to methylate full length YY1 *in vitro* (Figure 7A). Furthermore, immuno-pulldown experiment suggested *in vivo* interaction between FLAG-CARM1 and HA-YY1 in HEK293T cells (Figure 7B).

YY1 is known to interact with multiple coregulators in cell to modulate gene expression. For example, p300/CBP and PRMT1 are known coactivators of YY1 mediated gene regulation [37, 38]. Similarly, HDACs have been shown to function as corepressors for YY1 [39]. In the present study, to test the possibility of a role for CARM1 as a coactivator in the context of YY1 mediated gene regulation in cells, a reporter assay was performed using CARM1 promoter driven luciferase expression system. When CARM1 was exogenously overexpressed along with YY1 in HEK293T cells, the transactivation potential of YY1 was found to be enhanced, as indicated by increased luciferse activity (Figure 7C). The catalytically deficient mutant E267Q CARM1 showed similar activation of the luciferase reporter, indicating arginine methylation independent coactivator function of CARM1. This result also indicates a positive feedback loop for CARM1, where CARM1 regulates its own gene promoter via coactivation of YY1.

Analysis on the amino acid sequence of YY1 revealed that the DNA binding domain of YY1 alone harbors 11 arginine residues, where the rest of protein harbors only 3 arginine residues. Thus, the probability of YY1-DBD being the putative target for methylation by CARM1 seemed to be high. To test this possibility, *in vitro* methylation assay was performed with DBD of YY1. Indeed, CARM1 was able to methylate the DNA binding domain of YY1 *in vitro* (Figure 7D). Methylation of YY1-DBD by CARM1 was negatively affected in presence of a pharmacological inhibitor of CARM1 namely PRMT inhibitor V [40] (Figure 7E). In order to understand the effect of CARM1 in modulating DNA binding properties of YY1, EMSA was performed with YY1-DBD. CARM1 itself seemed to enhance DNA binding ability of YY1-DBD *in vitro*, which did not seem to vary much when arginine methylation was allowed in presence of methyl donor SAM (Figure 7F). Therefore, CARM1 might enhance DNA binding properties of YY1 independent of arginine methylation of YY1.

Mass spectrometry analysis was performed to identify the sites of arginine methylation on YY1 mediated by CARM1. CARM1 seemed to methylate YY1-DBD on R281, R294, R323, R342, R363 and R381 *in vitro*. The A-Score, localization probability and the number of spectra providing evidence for each site have been listed in Table 1. Future studies will be necessary to determine relative propensity as well as *in vivo* relevance of above mentioned sites for arginine methylation mediated by CARM1.

**Table 1:** The list of arginine residues on YY1 found to be methylated by mass spectrometry

S.N.	Sites	Modification	Best A score	Localization probability	SpC
1	R281	Methyl	169.11	1.000	3
2	R294	Methyl	211.02	1.000	3
3	R323	Methyl	188.24	1.000	11
4	R342	Methyl	46.21	1.000	1
5	R363	Methyl	168.56	1.000	1
6	R381	Methyl	1000.0	1.000	9
7	R381	Dimethyl	26.34	0.995	1

Methylation status, best A-Score, localization probability and the number of spectra providing evidence for each site have been indicated.

## DISCUSSION

CARM1 has been shown to be overexpressed in several cancer types such as colon cancer, prostate cancer and breast cancer [23–29]. In the present study CARM1 was also found to be upregulated in oral cancer patient tumor tissues. However, the mechanisms of transcriptional dysregulation in CARM1 expression in different pathophysiological conditions have not been addressed before. In this study we show that YY1 is a positive regulator CARM1 promoter. We also observed a positive correlation in protein expression of both YY1 and CARM1 in oral cancer tumor tissues. This observation indicates that YY1 mediated regulation of CARM1 gene promoter could be partly responsible for overexpression condition of CARM1 in oral cancer.

The roles of CARM1 and YY1 in the progression of oral carcinogenesis have not been explored before. In the present study investigation with silencing of both CARM1 and YY1 unraveled the oncogenic functions of both the proteins towards oral cancer manifestation. Inducible knock-down of CARM1 did not affect the proliferation ability of oral cancer cells; however it inhibited migration potential and colony formation by oral cancer cells. On the other hand, inducible silencing of YY1 resulted in reduced proliferation, reduced clonal propagation as well as slower migration of oral cancer cells. Transcriptomic analysis along with qRT-PCR validations suggested pro-proliferative, pro-angiogenic and pro-metastatic role of YY1. In this context, xenograft studies with silencing of YY1 clearly showed oncogenic role of YY1 *in vivo*.

YY1 was discovered to be a substrate of CARM1 mediated arginine methylation, where CARM1 could coactivate YY1 mediated reporter gene activation *in cellulo*. It would be necessary to characterize the sites of arginine methylation on YY1 that were identified from mass spectrometry. Although CARM1 could coactivate YY1 mediated transcription independent of its methyltransferase activity, study on the sites of arginine methylation would provide biochemical insight to understand the effect of the modification on molecular properties of YY1 as well as to understand different probable cross-talks with other post-translational modifications.

**Figure 8:**
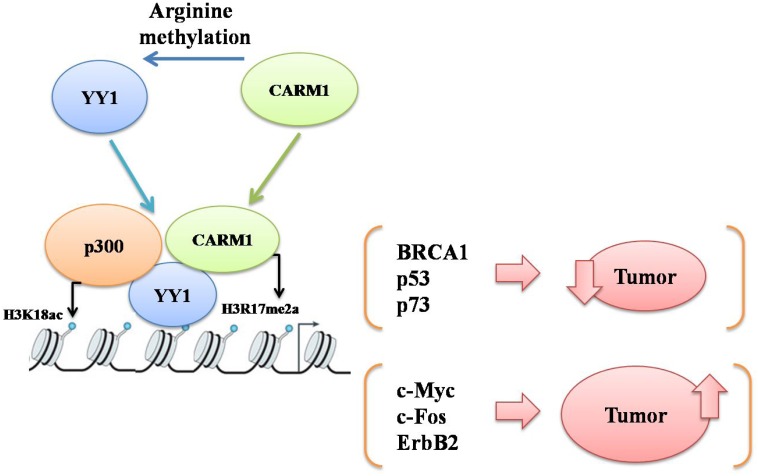
A schematic to explain probable mechanisms of cooperative action between YY1 and CARM1 either to suppress or promote carcinogenesis by upregulating either YY1 responsive tumor suppressor genes (such as BRCA1, p53 and p73) or YY1 responsive oncogenes (such as c-Myc, c-Fos and Erb B2) respectively in a context dependent manner.

Transcriptomic analysis with knock down of CARM1 in the context of oral cancer would be necessary to identify the genes which are regulated by both YY1 and CARM1. This would help us to distinguish the set of YY1 responsive genes which are CARM1 dependent in the context of oral cancer. It would be interesting to investigate whether YY1 employs CARM1 to regulate expression of both oncogenes as well as tumor suppressor genes in context dependent manner. As CARM1 has not been reported to possess corepressor activity, tumor suppressive YY1 might also utilize coactivator properties of CARM1 to activate tumor suppressor genes to inhibit tumor growth in certain cancer types (Figure 8).

Apart from assisting YY1 in regulating gene expression, CARM1 overexpression also implies that there would be increased availability of CARM1 for other cellular functions which may have significant implications in oral carcinogenesis that are independent of YY1 function. It could be via coactivation of transcription factors other than YY1, through chromatin remodeling function of CARM1 or even via modulation of RNA metabolism in cell. CARM1 is the only PRMT family member responsible for deposition of H3R17me2a modification on chromatin, which is known to favor transcriptional activation [41]. In the present study immunohistochemistry analysis reveals higher prevalence of H3R17me2a mark in oral cancer patient samples; a promising observation which might indicate the possibility of H3R17me2a modification to be considered as a diagnostic epigenetic mark in oral cancer in near future. Clinically relevant RNAi therapy and small molecule inhibitors could also be developed and employed targeting YY1 and CARM1 to suppress oral cancer growth in near future.

## MATERIALS AND METHODS

### Patient tumor samples

Oral cancer patient tumor tissues were obtained from HCG-BIO, Sri Devaraj Urs Medical college, Kolar, Bangalore and NEHU, Shillong, India. The clinico-pathological information available on the oral cancer patients have been summarized in Supplementary Table 1. The patients were of both the genders: males (60%) and females (40%) aged between 28–87 yrs. Out of 30 tumor samples, 22 (73.33%) were of grade I, 6 (20%) were of grade II and 2 (6.67%) were of grade III in cellular differentiation.

### Immunohistochemistry analysis

Tumor and adjacent normal tissues from oral cancer patients were collected in formalin from hospital on the day of surgery. The tissues were dehydrated, paraffin embedded, and sectioned with a microtome (Leica). The sectioned tissues were baked on glass slides at higher temperature followed by deparaffinization in xylene. The tissue sections were rehydrated with sequential immersion in graded alcohols (100%, 70%, 50% in PBS). The antigen retrieval was done by treating the rehydrated sections with low pH citrate buffer. Then peroxidase block with 3% H_2_O_2_ followed by blocking with 5% skimmed milk was carried out. The sections were incubated with different primary antibodies and then secondary antibodies in 1% skimmed milk. Tissue sections were incubated with Strept-Avidin Biotin kit (Dako) reagents. Immuno-reactivity (brown precipitate) was allowed to develop in the presence of DAB (Diaminobenzidine tetrahydrochloride) (Sigma), and counterstaining was done with Hematoxylin. The stained tissues were mounted with a cover slip with the help of DPX. The images of immune-stained tissues were taken with the help of microscope and H-scoring was performed. The oral cells were counted and based on the intensity of staining, scores were calculated as follows: 1 × percentage of weakly stained nuclei + 2 × percentage of moderately stained nuclei + 3 × percentage of strongly stained nuclei giving a range of 0 to 300 of H-score.

### Cell culture

HEK293T cell line was obtained from ATCC (American Type Culture Collection). AW8507 cell line (RRID: CVCL_D653, [45]) was obtained from ACTREC, Mumbai, India. Both HEK293T and AW8507 cell lines were grown in DMEM (Dulbecco’s Modified Eagle’s Medium). AW8507 cells containing Tet-ON-shYY1 and Tet-ON-shCARM1 (post transfection with respective plasmids) isolated with Puromycin selection and FACS for RFP positivity. In all cases the growth medium was supplemented with 10% FBS (Fetal Bovine Serum) for cell culture irrespective of the cell line. All the mammalian cell lines were grown in 37° C incubator with 5% CO_2_ and 90% relative humidity.

Luciferase assay with CARM1 promoter, Immunoblotting, total RNA isolation, qRT-PCR and ChIP-qPCR: Performed as described in *Behera AK et al., (2018) FEBS J.* (The nucleotide sequences of primers used in the study have been listed in Supplementary Table 3).

### Purification of FLAG-CARM1 from Sf21 cells

Sf21 cells were grown in TC100 medium in 25° C incubator. 6–7 million cells were seeded in each of 10, 150 mm dishes and infected with baculovirus containing CARM1 expression plasmid for 64–70 hrs. The infected cells were scraped off the plates to collect in PBS and harvested by centrifugation at 2000 rpm at 4° C. Cells were once washed with PBS followed by resuspension in lysis buffer (20 mM Tris-Cl, pH 7.4, 500 mM NaCl, 4 mM MgCl_2_, 2 mM EDTA, 2 mM DTT, 20 mM β-glycerophosphate, 0.4 mM PMSF, 20 % glycerol). Dounce homogenizer was used for effective lysis of cells (4 strokes, 6 times in 3 min intervals on ice). The sample post homogenization was diluted with dilution buffer (20 mM Tris-Cl, pH 7.4, 10% glycerol, 0.02% NP-40). The lysed cells were centrifuged at 16000 rpm for 30 min at 4° C and the resultant supernatant was incubated with M2 agarose beads for 3hrs at 4° C on end to end rotor. The beads were washed with wash buffer (20 mM Tris-Cl pH 7.4, 150 mM NaCl, 2 mM MgCl_2_, 0.2 mM EDTA, 1 mM DTT, 10 mM β-glycerophosphate, 0.2 mM PMSF, 0.01% NP40, 15% glycerol) for 4–5 times followed by elution of bound protein with elution buffer (BC100 with 150ug/ml FLAG peptide) at 4° C.

### Purification of His-YY1 from *E. coli*

*E. coli* BL21 cells transformed with bacterial expression vector of pET28b-His-YY1, were grown in LB medium and induced with 1 mM IPTG at cell density OD_600_ of 0.6. Induced cells were grown in 37° C for 3hrs, then pelleted, resuspended in Buffer A (6M Guanidine HCl, 25 mM Tris-Cl, pH 8.0, 100 mM NaCl, 5 mM βME, 10 mM Imidazole) and sonicated. Centrifugation of the lysed cells was done at 16,000 rpm for 30 minutes at 4° C. Supernatant was incubated with Ni-NTA beads for 3hrs at 4° C with constant rotation. Beads were washed with Buffer A with 30 mM imidazole for 5–6 times. Elution was done with Buffer A containing 400 mM Imidazole. Eluted proteins were pulled together and dialyzed against Refolding buffer (25 mM Tris-Cl/50 mM HEPES pH 7.8, 10 mM MgCl_2,_ 100 mM NaCl, 5 mM DTT and 0.1 mM ZnCl_2_). The dialysate was centrifuged and supernatant containing purified His-YY1 was stored.

### Purification of His-YY1-DBD (aa 223-414) from *E. coli*

*E. coli* BL21 cells harboring pET28b-His-YY1-DBD expression plasmid were grown in 100 ml of LB medium at 37° C for overnight, which was scaled up to 1L the next day. The optical density of bacterial culture was measured at repeated intervals at 600 nm of wave length and when absorbance value reached 0.6, bacterial cells were induced with 0.5 mM IPTG for 3hrs at 37° C. The bacterial culture was centrifuged to harvest the cells. The bacterial cells were resuspended in lysis buffer (20 mM Tris-Cl, pH 7.5, 20% glycerol, 0.2 mM EDTA, 0.3M KCl, 0.1% NP40, 20 mM Imidazole, 2 mM PMSF, and 2 mM β-ME) and sonicated in order to lyse the cells. The lysed cells in buffer were centrifuged at 16000 rpm for 30 mins at 4° C. The resulting supernatant was incubated with Ni-NTA beads for 3hrs at 4° C on end to end rotor. The beads were washed once with lysis buffer and 4–5 times with wash buffer (20 mM Tris-Cl, pH 7.5, 20% glycerol, 0.2 mM EDTA, 0.3M KCl, 0.1% NP40, 40 mM Imidazole, 2 mM PMSF and 2 mM β-ME). The protein bound to beads were eluted with elution buffer (20 mM Tris-Cl, pH 7.5, 20% glycerol, 0.1 mM EDTA, 2 mM PMSF, 0.5M KCl, 0.2% NP40, 300 mM imidazole and 1 mM β-ME) at 4° C.

### *In vitro* methylation assay

Recombinant full length enzyme FLAG-CARM1 and recombinant substrates were incubated in methyltransferase buffer (20 mM Tris-HCl, pH 8.0, 4 mM EDTA, 200 mM NaCl) with S-adenosyl-L-[methyl-^3^H]methionine (15 Ci/mmol, NEN-Perkin Elmer) or unlabelled SAM according to the necessity of the experiment, at 30° C for desired period of time. Reaction mixture was run on 12% SDS PAGE. Gel was Coomassie stained and destained and processed for autoradiography.

### Electrophoretic mobility shift assay

Target DNA oligonucleotides from CARM1 promoter (Listed in Supplementary Table 3) were end labelled with γ^32^P ATP using T4 Polynucleotide kinase and isolated via Phenol-chloroform-isopropanol (PCI) extraction method. The aqueous phase obtained from PCI separation was incubated with absolute ethanol in presence of sodium acetate and stored in –80° C for 1hr. The labeled oligonucleotides were obtained in the form of a pellet upon centrifugation at 16000 rpm. The labeled single strand DNA pellet was air dried and reconstituted in annealing buffer (10 mM Tris-Cl, pH 8.0, 20 mM NaCl) with addition of equimolar amounts of complementary strand. The annealing was allowed to occur at 85° C with gradual cooling to RT. The annealed double strand probes were purified by passing through Sephadex C-50 column. The activity of radiolabelled probes was measured with the help of scintillation counter. Labelled oligonucleotides with 5000 cpm were incubated with respective recombinant proteins at 30° C for 30 minutes in EMSA buffer (10% glycerol, 75 mM KCl, 10 mM HEPES, 5 mM MgCl_2_) and complexes were resolved on 5% native PAGE (5% acrylamide: bisacrylamide (19:1) mix, 0.5X TBE, 0.1% APS, 0.03% TEMED) for 2 hrs at 4° C. Gel was dried and kept for exposure to X-ray films.

### Immuno-pulldown assay

HEK293T cells were transfected with pcDNA3-HA-YY1 and pCMV-FLAG-CARM1 plasmids. After 24 h, adherent cells were lysed with lysis buffer (50 mM Tris-Cl, pH 7.4, 150 mM NaCl, 1 mM EDTA, 1% Triton-X 100) on a rocker at 4° C for 30 minutes. Samples were collected in centrifuge tubes and kept on ice for 15 minutes and then subjected to centrifugation at 2000rpm for 15 minutes at 4° C. Supernatant was incubated with M2 agarose beads (equilibrated with lysis buffer) for 6 h at 4° C on a rotating rocker. Beads were collected with centrifugation at 2000 rpm for 10 minutes at 4° C. Beads were washed with lysis buffer (once) and wash buffer (20 mM Tris-Cl, pH 7.4, 150 mM NaCl, 2 mM MgCl2, 1 mM DTT, 0.2 mM EDTA, 0.01% NP-40, 0.2 mM PMSF, 10 mM β-Glycerophosphate, 15% Glycerol) several times. Elution was performed with BC100 containing 200 ng/ul of FLAG peptide. Immunoblotting was performed with HA and FLAG tag-specific antibodies.

### MTT assay

Cells were seeded in 96 well plates and maintained under desired conditions. On the respective days cells were treated with MTT [3-(4,5-dimethylthiazol-2-yl)-2,5-diphenyltetrazolium bromide] at the final concentration of 0.5 mg/ml in growing media for 2 hrs to allow intracellular reduction of above mentioned tetrazolium dye (yellow) to insoluble formazan (purple) by oxidoreductase enzymes. Then the media was aspirated and the cells were added with DMSO (Dimethyl Sulfoxide) as the solubilization agent for formazan. After 15 minutes, the colored solution (solubilized formazan in DMSO) was saved and taken for colorimetric quantification. Absorbance was measured at OD of 570 nm with the help of a multiwell scanning spectrophotometer (ELISA reader). The degree of light absorption (absorbance values) were plotted to correlate with cellular number as indirect representation of cellular proliferation.

### Clonogenic assay

The cells were seeded in 6 well plates (~500 per well) with desired conditions in triplicates and allowed to grow for 10 days. The colonies of cells thus produced, were fixed with ice cold methanol for 25 minutes followed by staining with 0.5% crystal violet solution prepared in methanol, for 2 hrs. The colonies of cells were washed with PBS, dried and then processed for imaging as well as estimation of colony number.

### *In vitro* wound healing assay

Cells were seeded in 30 mm dishes and grown to confluency. A straight scratch was produced on the monolayer of cells with the help of a microtip. Then live cell imaging of cells was performed with the microscope (Axiovert 200M) focused on the scratch for 24 hrs, where images were taken at intervals of 5 minutes to monitor closure of scratch as a reflection of cellular migration.

### Xenograft study

All procedures involving animals were reviewed and approved by the NUS Institutional Animal Care and Use Committee. NOD-SCID gamma (NSG) mice (7 week old) were purchased from InVivosPte Ltd. (Singapore). The mice were implanted with either AW8507_Tet-ON-shYY1 or AW8507_Tet-ON-shCARM1 (1 × 10^6^ cells) mixed with 10% matrigel matrix (BD Biosciences, USA) subcutaneously on the flank region. Doxycycline hyclate treatment was started when the size of the tumor was 0.2 to 0.3 cm. The mice were given either plain water (vehicle control) or doxycycline (200 μg/ml) and 1% sucrose mixed in drinking water for seven weeks. The tumor size and body weight of the mice was measured once a week for the duration of the experiment. The tumor volume was calculated using the formula [L × W^2^]/2, where W and L are the width (short diameter) and the length (long diameter) of the tumor respectively.

### Reagents, plasmids and antibodies

TC100 insect medium (Ref: IML007), PBS (Ref: TL1006), Trypsin-EDTA solution 10× (Ref: TCL070) and Antibiotic antimyotic solution 100× (Ref: A002A) were obtained from HIMEDIA. The lipofectamine used for transfection was obtained from Invitrogen (Ref: 11668019). DMEM powder (Ref: D1152), MTT (Ref: M5655) and SAM (Ref: A7007) were obtained from Sigma-Aldrich (St. Louis, Missouri, USA). TRIzol reagent (Ref: 15596018) was obtained from ambion life technologies. The TRIPZ-shRNA plasmids used for stable cell line generation were obtained from Dharmacon (Ref: CARM1 sh -V3THS_319980 and YY1 sh-V2THS_219592). The commercial antibodies used in the study include Tubulin from calbiochem (Ref: CP06), CARM1 from abcam (Ref: Ab84370), YY1 from abcam (Ref: Ab12132) and H3R17me2a from Millipore (07–214). Tritiated SAM was obtained from Perkin-Elmer (Ref: NET155250UC). Luciferase assay buffer (Ref: E151A) and reporter lysis buffer (Ref: E397A) were purchased from Promega (Madison, Wisconsin, USA). CARM1 inhibitor (PRMT inhibitor V) was purchased from Calbiochem-Millipore (Ref: 217531).

### Transcriptomic analysis by microarray

Total RNA was isolated from cells grown under different assay conditions. The quality and quantity of RNA was assessed spectrophotometrically by a Nanodrop and Bioanalyzer. Equal amounts of RNA samples were used to prepare cDNA library and processed for transcriptomic analysis using Illumina Gene Expression arrays. The raw data obtained from hybridization to illumina HT-12 array, was quantile normalized followed by baseline transformation to median of all the samples using GeneSpring GX 12.5 software. The differentially expressed genes were identified across the samples using volcano plot with a fold change threshold of 1.5 and *t* test *p* value threshold adjusted for false discovery rate less than 0.001 for statistical significance. Hierarchical clustering of differentially expressed genes in treated vs. control conditions was done using Euclidian algorithm with Centroid linkage rule to identify gene clusters whose expression levels were significantly reproduced across the replicates. Differentially expressed genes were subjected to biological significance analysis by GOElite tool to determine enriched biological pathways. Over representation Analysis (ORA) of significant biological categories (GeneOntology and Pathway) involving differentially expressed transcripts was performed and a network was modeled. GEO accession number for the microarray data: GSE125317.

### Mass spectrometry analysis

*In vitro* methylated His-YY1-DBD by FLAG-CARM1 in presence of SAM was separated on 12% SDS-PAGE. The destained gel bands containing the methylated targeted protein were washed with 25 mM ammonium bicarbonate followed by acetonitrile. Proteins were reduced with 10 mM dithiothreitol at 60° C followed by alkylation with 50 mM iodoacetamide at RT. Proteins were subjected to multi-enzyme digestion as follows: trypsin (Promega) for 4h, chymotrypsin/elastase (Promega) for 12 h at 37° C followed by Quenching with formic acid. Each gel digest was analyzed by nano LC/MS/MS with a Waters NanoAcquity HPLC system interfaced to a ThermoFisher Q Exactive at MS Bioworks, LLC (Ann Arbor, MI). Peptides were eluted through a 75 μm analytical column of Luna C18 resin (Phenomenex) at 350 nL/min. The mass spectrometer was operated in data-dependent mode. MS and MS/MS were performed in the Orbitrap at 70,000 FWHM and 17,500 FWHM resolutions respectively. The fifteen most abundant ions were selected for MS/MS. Byonic mzID files were parsed into Scaffold software to validate and filter and to create a nonredundant list per sample. Data were filtered using a minimum peptide value of 50% (Prophet scores) and a minimum protein value of 95% requiring at least two unique peptides per protein.

### Bioinformatic analysis with TCGA RNA-seq data

RSEM normalized expression data pertaining to CARM1 and YY1 was downloaded from www.cbioportal.org. The TCGA RNA-Seq data corresponding to 32 different tumor types was plotted as a box-whisker plot using R software [42]. To further interpret the expression status of the two genes, PCA analysis using two different R packages [43, 44] was carried out to study the correlation between the expression patterns of the genes across different cancers.

### Statistical analysis

All the statistical analyses were performed using GraphPad Prism 7 Software (California, USA). The data obtained from three independent experiments were expressed as mean ± SEM. Two tailed unpaired Student’s *t*-test was used to determine the statistical significance values. A *p*-value of equal to or less than 0.05 was considered statistically significant.

## SUPPLEMENTARY MATERIALS



## Abbreviations

BCl2: B-cell lymphoma 2
BRCA1: Breast cancer 1
CARM1: Coactivator associated arginine methyltransferase
DR: Death Receptor
ESCC: Esophageal squamous cell carcinoma
MMP: Matrix metalloproteinase
MEF2C: Myocyte enhancer factor-2C
OSCC: Oral squamous cell carcinoma
PRMT: PGL: Placental growth factor, Protein arginine methyltransferase
SAM: S-adenosyl- L-methionine
TGF: Transforming growth factor
TNF-α: Tumor necrosis factor alpha
TRAIL: TNF-related apoptosis-inducing ligand
VEGFC: Vascular Endothelial Growth Factor C
VEGFR: Vascular Endothelial Growth Factor Receptor
YY1: Ying Yang 1
