# Supplementary material for: Functional interplay between YY1 and CARM1 promotes oral carcinogenesis
Source: Oncotarget. 2019 Jun 4;10(38):3709–24. doi: 10.18632/oncotarget.26984 (PMC6557205; doi:10.18632/oncotarget.26984)
Supplement: Supplementary file 1 [file oncotarget-10-3709-s001.pdf]

Functional interplay between YY1 and CARM1 promotes oral carcinogenesis

SUPPLEMENTARY MATERIALS

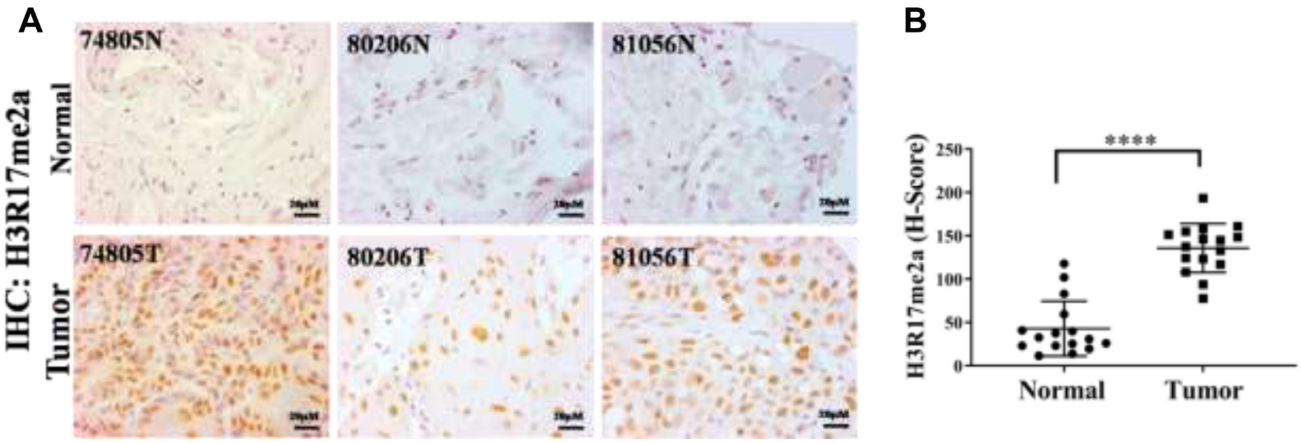

Supplementary Figure 1: H3R17me2a mark is upregulated in oral tumor tissues. (**A**) Representative immunohistochemistry images of paired oral cancer patient samples stained with H3R17me2a antibody. The numbers on images represent patient ID (e.g., 74805, 80206 and 81056). Image scale: 20 µm. (**B**) H-scoring for H3R17me2a staining in oral cancer patient tumor tissue compared to adjacent normal tissue (Student's *t*-test: $^{****}p < 0.0001$, $n = 16$).

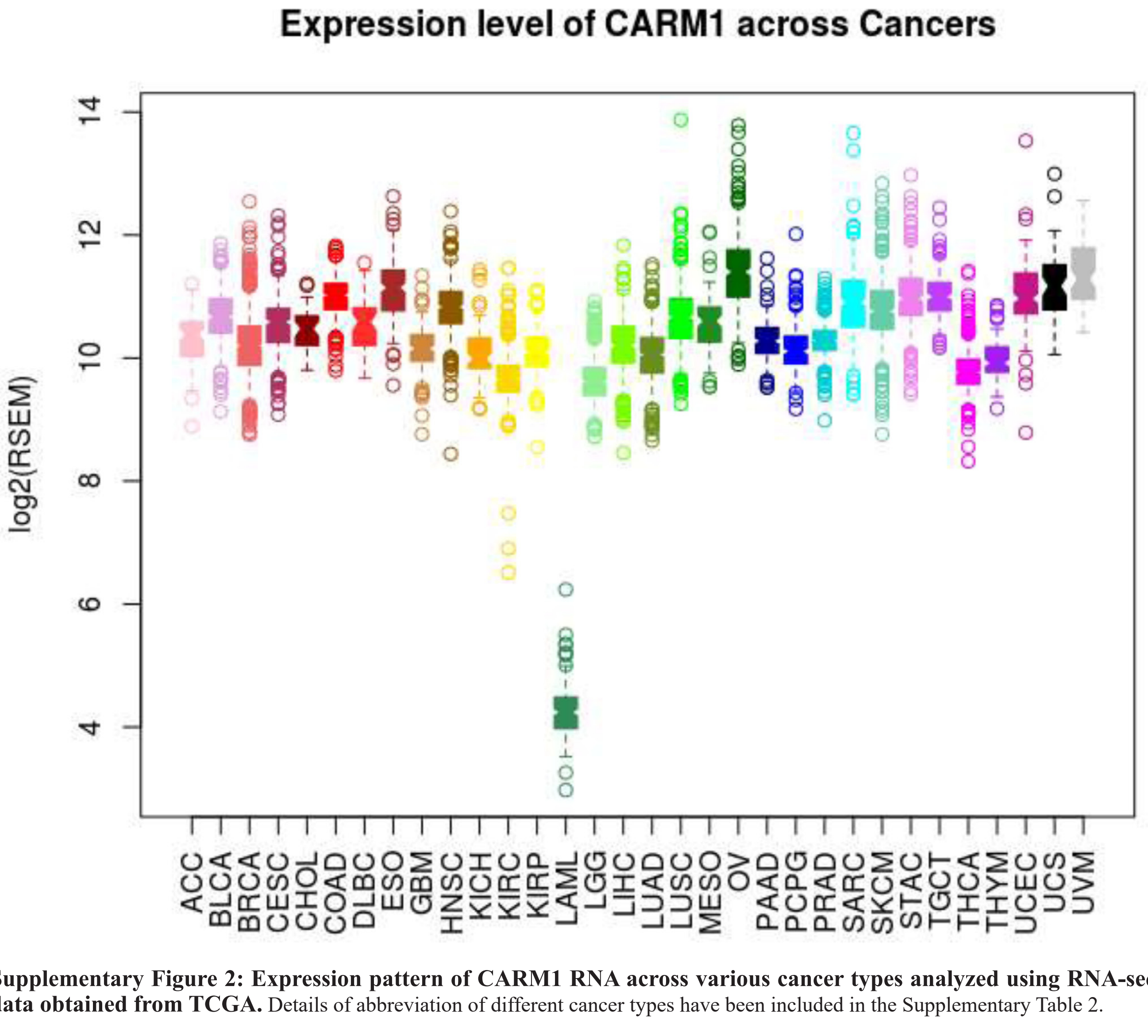

Supplementary Figure 2: Expression pattern of CARM1 RNA across various cancer types analyzed using RNA-seq data obtained from TCGA. Details of abbreviation of different cancer types have been included in the Supplementary Table 2.

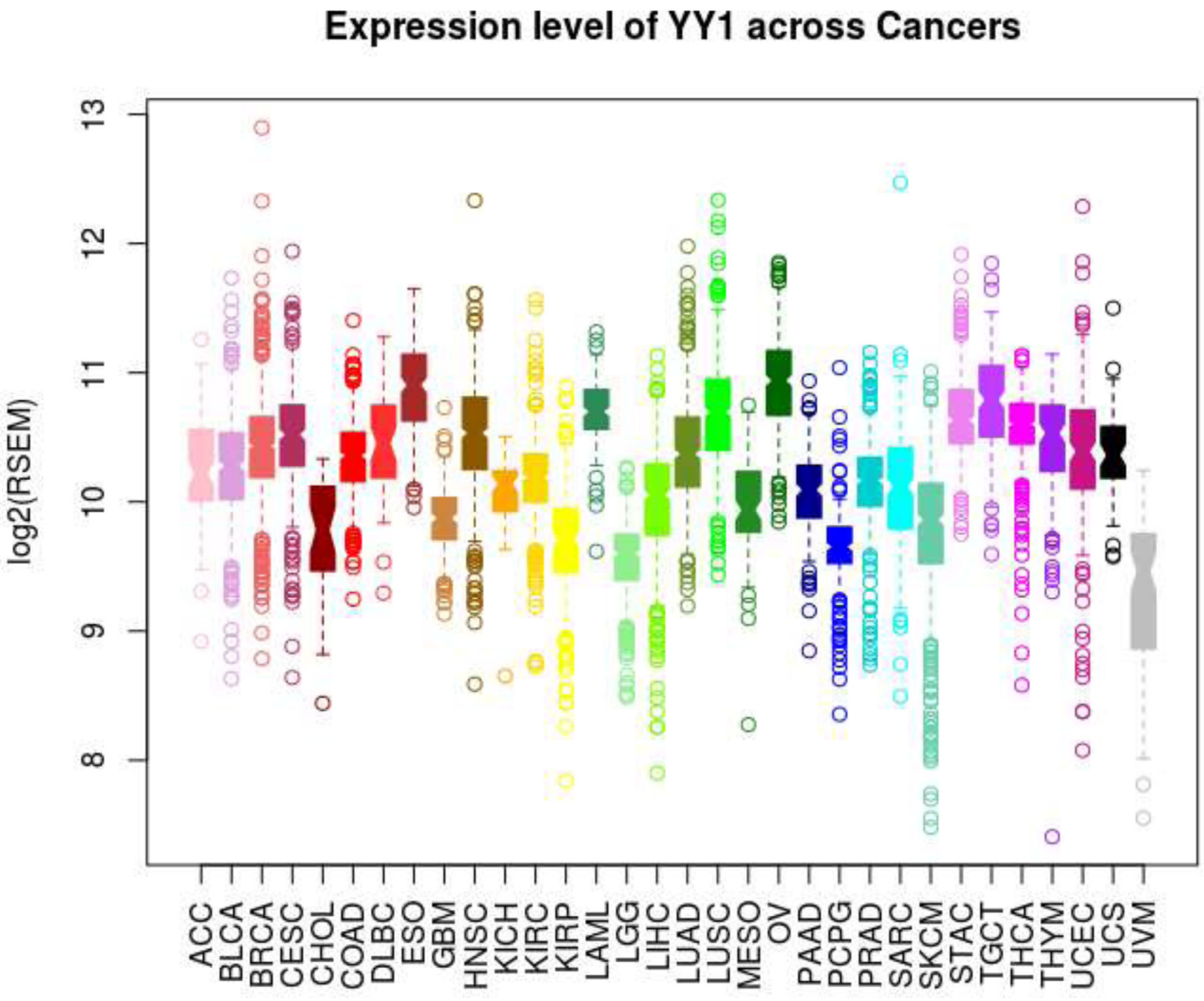

Supplementary Figure 3: Expression pattern of YY1 RNA across various cancer types analyzed using RNA-seq data obtained from TCGA. Details of abbreviation of different cancer types have been included in the Supplementary Table 2.

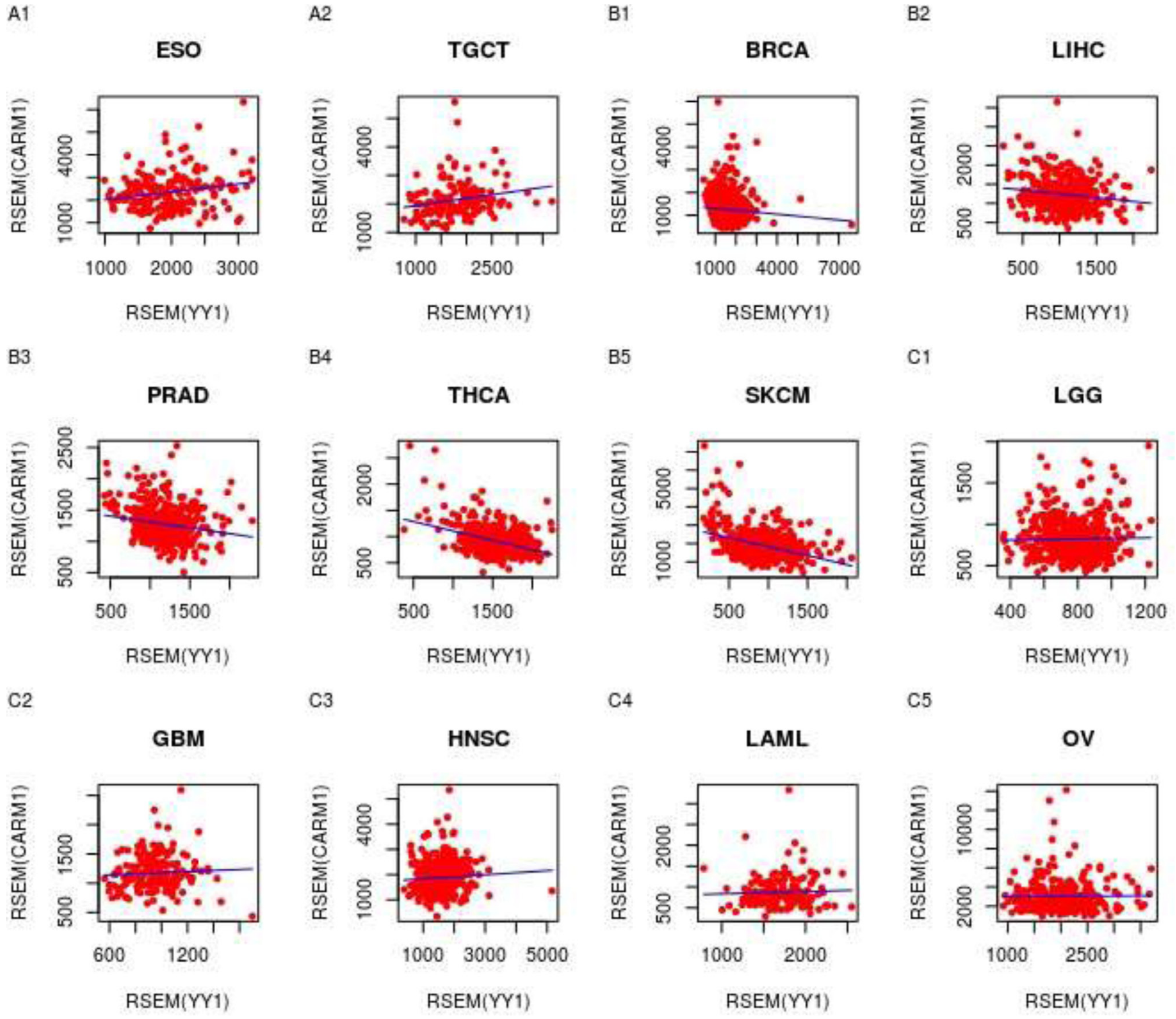

Supplementary Figure 4: Plots depicting PCA (Pearson's correlation analysis) between RNA expression of CARM1 and YY1 using RNA-seq data obtained from TCGA. ESO and TGCT showed positive correlation (A1 and A2); BRCA, LIHC, PRAD, THCA and SKCM showed negative correlation (B1-B5); LGG, GBM, HNSC, LAML and OV showed no correlation (C1-C5). Details of abbreviation of different cancer types have been included in the Supplementary Table 2.

Supplementary Table 1: Clinico-pathological information on the oral cancer patients

						H score	H-score	H-score
**S.N.**	**Patient ID**	**Gender**	**Age**	**Differentiation**	**Grade**	**CARM1 (T/N)**	**YY1 (T/N)**	**H3R17me2a (T/N)**
1	63	F	48	Well differentiated	I	112.34/78.73	137.51/118.62	NA
2	65	M	45	Well differentiated	I	133.96/55.18	123.51/56.75	NA
3	188	F	60	Well differentiated	I	115.99/65.9	NA	NA
4	211	M	45	Well differentiated	I	122.94/59.93	201.58/101.18	NA
5	215	M	70	Well differentiated	I	148.96/59.42	191.08/141.07	NA
6	224	F	70	Well differentiated	I	134.94/59.05	173.66/86.2	NA
7	243	M	43	Well differentiated	I	149.87/97.85	121.9/81.16	NA
8	268	M	60	Well differentiated	I	161.96/98.63	145.82/93.38	NA
9	439	M	29	Well differentiated	I	109.88/31.78	122.15/21.32	NA
10	532	M	44	Well differentiated	I	153.65/18.27	90.64/16.63	NA
11	626	M	28	Well differentiated	I	168.96/103.11	NA	NA
12	946	M	50	Well differentiated	I	151.93/105.09	160/60.51	NA
13	66911	M	40	Moderately differentiated	II	111.41/22.9	150.36/69.71	137.6/40.65
14	66951	M	NA	Well differentiated	I	NA	140.4/60.8	146.14/13.85
15	70531	M	87	Well differentiated	I	NA	105.16/38.01	77.62/19.43
16	74597	M	51	Poorly differentiated	III	64.71/5.95	96.59/62.61	NA
17	74805	F	37	Poorly differentiated	III	104.89/13.86	173.69/87.07	158.07/25.91
18	79962	F	50	Moderately differentiated	II	21.84/3.46	NA	NA
19	80206	M	43	Well differentiated	I	86.66/5.32	172.37/76.4	151.36/11.44
20	81056	M	54	Moderately differentiated	II	58.44/16.36	191.15/102.89	144.99/30.67
21	81673	F	60	Well differentiated	I	NA	23.31/32.03	93.87/23.08
22	81908	F	55	Well differentiated	I	2.62/1.11	12.52/19.28	116.93/83.12
23	82972	M	50	Moderately differentiated	II	NA	11.08/11.04	148.48/32.66
24	84429	F	60	Well differentiated	I	9.84/0.69	29.74/34.07	154.91/102.02
25	921188	F	60	Well differentiated	I	11.7/2.03	46.78/5.51	123.3/39.71
26	924073	F	60	Well differentiated	I	134.18/3.04	71.52/11.05	193.43/59.38
27	924178	F	65	Moderately differentiated	II	13.35/2.19	29.32/12.49	108.1/23.09
28	930713	F	60	Well differentiated	I	45.13/25.25	18.04/12.49	160.91/117.78
29	934927	M	66	Moderately differentiated	II	8.74/3.86	16.86/27	132.84/37.4
30	998486	M	58	Well differentiated	I	143.07/3.25	60.8/8.4	124.36/25.26

No of patients: 30
Age: 28-87
Gender:
1. Males: 18 (60%)
2. Females: 12 (40%)

Cell differentiation /Grade:
1. Well differentiated (Grade I): 22 (73.33%)
2. Moderately differentiated (Grade II): 6 (20%)
3. Poorly differentiated (Grade III): 2 (6.67%)

Abbreviations: NA: Not available; T: Tumor; N: Normal.

Supplementary Table 2: Summary on the PCA (Pearson's correlation analysis) between RNA expression of CARM1 and YY1 across different cancer types using RNA-seq data obtained from TCGA

Tumor	Number of Samples	Pearson's Correlation Value	*p*-value	Tumor Type
**Positive Correlation**				
ESO	185	0.21	0.0034	**Esophageal Carcinoma**
TGCT	156	0.21	0.0072	**Testicular Germ Cell Cancer**
**Negative Correlation**				
BLCA	406	-0.13	0.0064	**Bladder Urothelial Carcinoma**
BRCA	1100	-0.076	0.0117	**Breast Invasive Carcinoma**
KIRC	534	-0.27	1.75E-010	**Kidney Renal Cell Clear Carcinoma**
LIHC	373	-0.15	0.0033	**Liver Hepatocellular Carcinoma**
MESO	87	-0.3	0.0045	**Mesothelioma**
PAAD	179	-0.25	0.0006	**Pancreatic Adenocarcinoma**
PCPG	184	-0.27	0.0001	**Pheochromocytoma and Paraganglioma**
PRAD	498	-0.19	2.97E-005	**Prostate Adenocarcinoma**
SKCM	472	-0.38	< 2.2E-016	**Skin Cutaneous Melanoma**
THCA	509	-0.4	< 2.2E-016	**Thyroid Carcinoma**
THYM	120	-0.38	1.45E-005	**Thymoma**
UVM	80	-0.73	1.77E-014	**Uveal Melanoma**
**No Correlation**				
ACC	79	0.033	0.7699	**Adrenocortical Carcinoma**
CESC	306	0.023	0.6936	**Cervical Squamous Cell Carcinoma and Endocervical Adenocarcinoma**
CHOL	36	-0.11	0.5045	**Cholangiocarcinoma**
COAD	382	-0.058	0.2545	**Colorectal Adenocarcinoma**
DLBC	48	0.25	0.0908	**Lymphoid Neoplasm Diffuse Large B-cell Lymphoma**
GBM	166	0.56	0.4766	**Glioblastoma Multiforme**
HNSC	522	0.069	0.1175	**Head and Neck Squamous Cell Carcinoma**
KICH	66	0.18	0.1488	**Kidney Chromophobe**
KIRP	291	-0.066	0.2615	**Kidney Renal Papillary Cell Carcinoma**
LAML	173	0.043	0.5751	**Acute Myeloid Leukemia**
LGG	530	0.022	0.618	**Brain Lower Grade Glioma**
LUAD	517	-0.047	0.2901	**Lung Adenocarcinoma**
LUSC	501	-0.0087	0.8458	**Lung Squamous Cell Carcinoma**
OV	307	0.0099	0.8622	**Ovarian Serous Cystadenocarcinoma**
SARC	263	0.0028	0.9645	**Sarcoma**
STAC	415	0.009	0.855	**Stomach Adenocarcinoma**
UCEC	177	-0.049	0.5174	**Uterine Corpus Endometrial Carcinoma**
UCS	57	0.15	0.2528	**Uterine Carcinosarcoma**

Supplementary Table 3: The list of oligos/primers used in the study with their respective nucleotide sequences

EMSA oligos	
CARM1proYY1_BS1 F	5′-GGGACCTCTGGATCCCATGGTTGGATGGCTGGGGGGTGGA-3′
CARM1proYY1_BS1 R	5′- TCCACCCCCCAGCCATCCAACCATGGGATCCAGAGGTCCC-3′
QPCR primers	
YY1 FP	5'-TGGTCCTCAGATGAAAAAAAAGATATTGAC-3′
YY1 RP	5'-GGCTTCATTCTAGCAAATTCTGCC-3′
CARM1 FP	5′-TTGATGTTGGCTGTGGCTCTGG-3′
CARM1 RP	5′-ATGGGCTCCGAGATGATGATGTCC-3′
PGF FP	5′- CAGAGGTGGAAGTGGTACCC-3′
PGF RP	5′- AGTGCAGATTCTCATCGCCG-3′
VEGFC FP	5′- TCATGAGTTCATCTACACTGGACA-3′
VEGFC RP	5′- CCGGACTCGACCTCTCG-3′
VIM FP	5′- AATGGCTCGTCACCTTCGTG-3′
VIM RP	5′- CAGATTAGTTTCCCTCAGGTTCAG-3′
TWIST1 FP	5′- GAGCGGCTCAGCTACGC-3′
TWIST1 RP	5′- TTCTCTGGAAACAATGACATCTAGGT-3′
MMP7 FP	5′- GAGTGAGCTACAGTGGGAACA-3′
MMP7 RP	5′- CTATGACGCGGGAGTTTAACATTC-3′
BCL3 FP	5′- TCGACGCAGTGGACATTAAGAG-3′
BCL3 RP	5′- ACATTTGCGCGTTCACGTT-3′
ACTB FP	5′-AGATGTGGATCAGGAAGCAGGA-3′
ACTB RP	5′-TCCTCGGCCACATTGTGAACTTTG-3′
ChIP-QPCR primers	
CARM1 pro_YY1ChIP_FP	5'-AACACGGGTCAAGGTGTTCCC-3′
CARM1 pro_YY1ChIP_RP	5'-CCCTGACTGCCCTTGAGAGATG-3′
